# Vitamin D-binding protein in cervicovaginal fluid as a non-invasive predictor of intra-amniotic infection and impending preterm delivery in women with preterm labor or preterm premature rupture of membranes

**DOI:** 10.1371/journal.pone.0198842

**Published:** 2018-06-07

**Authors:** Song Yi Kook, Kyo Hoon Park, Ji Ae Jang, Yu Mi Kim, Hyunsoo Park, Se Jeong Jeon

**Affiliations:** 1 Department of Obstetrics and Gynecology, Seoul National University College of Medicine, Seoul National University Bundang Hospital, Seongnam, Korea; 2 Center for High Risk Pregnancy and Neonate, Seoul National University Bundang Hospital, Seongnam, Korea; University of Illinois at Urbana-Champaign, UNITED STATES

## Abstract

**Objective:**

To determine whether vitamin D-binding protein (VDBP) in cervicovaginal fluid (CVF) is independently predictive of intra-amniotic infection and imminent spontaneous preterm delivery (SPTD, delivery within 48 hours) in women with preterm labor with intact membranes (PTL) or preterm premature rupture of membranes (PPROM).

**Method:**

This was a single-center retrospective cohort study. CVF samples for VDBP assays were obtained along with serum C-reactive protein (CRP) levels immediately after amniocentesis in consecutive women with PTL (n = 148) or PPROM (n = 103) between 23.0 and 34.0 weeks of gestation. VDBP levels in CVF were determined by enzyme-linked immunosorbent assay kits. The primary outcome measures were intra-amniotic infection [defined as positive amniotic fluid (AF) culture] and SPTD within 48 hours after sampling.

**Results:**

In the multivariable analysis, elevated VDBP levels in CVF samples of PTL women were significantly associated with intra-amniotic infection and imminent preterm delivery, even after adjusting for potential confounders (e.g., gestational age at sampling, parity, and serum CRP). However, these relationships were not found in women with PPROM. In women with PTL, the areas under receiver operating characteristic curves of CVF VDBP level for predicting intra-amniotic infection and imminent preterm delivery were 0.66 and 0.71, with cut-off values of 1.76 μg/mL (sensitivity of 64.3% and specificity of 78.4%) and 1.37 μg/mL (sensitivity of 65.4% and specificity of 72.6%), respectively. The CVF VDBP levels were significantly higher in women with PPROM than in those with PTL.

**Conclusions:**

VDBP in the CVF independently predicts intra-amniotic infection and imminent preterm delivery in women with PTL, whereas in women with PPROM, an elevated VDBP level in CVF is not associated with increased risks of these two outcome variables.

## Introduction

Spontaneous preterm labor and intact membranes (PTL) or spontaneous preterm premature rupture of the membranes (PPROM) accounts for approximately 70 to 80% of all preterm births, and preterm birth, owing to these causes in particular, is strongly associated with significant neonatal morbidity, mortality, and long-term disability [1–3]. Evidence suggests that the impact of preterm birth on adverse neonatal outcomes is directly related to the degree of prematurity and the occurrence of subclinical intra-uterine infection [4–6]. Therefore, the ability to predict the risk of spontaneous preterm delivery (SPTD) and intra-uterine infection more precisely, especially using non-invasive methods, has important clinical implications in terms of the treatment strategy (e.g., administration of medications [i.e., corticosteroid, antibiotics, and magnesium for neuroprotection] and transfer to a tertiary center) and the counseling of patients with PTL or PPROM.

Traditionally, measurement of inflammatory biomarkers in amniotic fluid (AF) sample obtained by amniocentesis has been extensively used for the prediction of intra-amniotic infection and SPTD. However, this measurement is currently limited in clinical practice due to the requirement of invasive AF sampling. In this context, cervicovaginal fluid (CVF), which can be obtained via noninvasive or minimally invasive methods, is a feasible alternative to the AF, because several studies have demonstrated changes in various inflammatory proteins present in the CVF in association with intra-amniotic infection/inflammation, premature ripening, cervical dilatation, and preterm birth [7–11].

Vitamin D-binding protein (VDBP) is a 58-kDa protein of the albumin superfamily that is mainly synthesized by hepatocytes. The established functions of VDBP include acting as a major carrier protein for vitamin D and its metabolites in serum, sequestering actin, and potentially modulating the inflammatory and immune response, and it is associated with the clinical progression of many diseases [12]. In particular, previous studies by Liong et al. and Hitti et al. that used the proteomic and cohort approaches have shown significantly increased expression of CVF VDBP in association with the occurrence of impending PPROM in asymptomatic women and of SPTD and intra-amniotic infection in women presenting with symptoms of PTL [11, 13–15]. However, these findings have not been confirmed by other studies. Moreover, whether the change in VDBP level in the CVF is associated with intra-amniotic infection and impending SPTD in women with PPROM remains unclear. Hence, the aim of this study was to determine whether the level of VDBP in CVF samples is independently predictive of intra-amniotic infection and SPTD within 48 hours in women with PTL or PPROM.

## Materials and methods

This retrospective cohort study was carried out at Seoul National University Bundang Hospital (Seongnam, Republic of Korea) from November 2008 to September 2015. The ethics committee at Seoul National University Bundang Hospital approved the study (IRB no. B-1105/128-102). The study population consisted of consecutive singleton pregnant women diagnosed with either spontaneous PTL (n = 148) or PPROM (n = 103) at 23 +0 to 34 +0 weeks of gestation. The inclusion criteria were as follows: (1) a live fetus was delivered; (2) an aliquot of CVF sample available for analysis; (3) amniocentesis performed to determine the microbial and inflammatory status of the AF at the time of enrollment; and (4) CVF collected at the time of amniocentesis. The exclusion criteria were multiple pregnancies, major fetal congenital anomalies, prior cervical cerclage, evidence of clinical chorioamnionitis at the time of presentation, and active labor at admission (defined as cervical dilatation greater than 3 cm by sterile speculum examination) in cases of PPROM. The diagnosis of PPROM was made on the basis of clinical findings of either a pool of AF in the posterior fornix or leakage of fluid through the cervix on sterile speculum examination, and a positive nitrazine test. PPROM was defined as the spontaneous rupture of membranes occurring prior to 37 weeks of gestation and before the onset of uterine contractions. Preterm labor was defined as the presence of regular uterine contractions, with a frequency of at least two contractions every 10 minutes, and cervical change that required hospitalization. Gestational age was calculated based on the last menstrual period and the first trimester or second trimester (≤20 weeks) ultrasound results, when available. During the study period, amniocentesis for retrieval of the AF and CVF sampling were immediately offered to patients who were admitted with either PTL or PPROM at our institution. The patients provided written informed consent for the collection and use of the CVF samples for research purposes. The primary outcome measures were a positive AF culture result and SPTD within 48 hours of sampling.

After informed consent was obtained, transabdominal amniocentesis was performed under sonographic guidance. The AF samples were then immediately sent to the laboratory for culturing of aerobic/anaerobic bacteria and genital mycoplasmas (*Ureaplasma urealyticum* and *Mycoplasma hominis*), the methods for which were previously described in detail [16]. At the time of amniocentesis, the CVF samples were obtained from all participants. The methods of CVF sample collection, processing, and storage have been previously described in detail [7]. In short, the CVF samples were collected from the posterior vaginal fornix using two sterile Dacron swabs (Puritan Medical, Guilford, ME, USA) placed for 15 seconds to absorb the cervicovaginal secretions under sterile speculum examination. The two Dacron swabs were then placed in two cryotubes each containing 1 mL of sample buffer and stored at -70°C for further analysis. Serum C-reactive protein (CRP) level was usually measured within 2–3 hours of sampling using latex-enhanced turbidimetric immunoassay (Denka Seiken, Tokyo, Japan), according to hospital protocol.

The CVF VDBP levels were measured using an enzyme-linked immunosorbent assay human DuoSet Kit (R&D Systems, Minneapolis, MN, USA) according to the manufacturer’s instruction. The VDBP quantification was validated for CVF samples by spike/recovery and linearity testing, which was performed in accordance with the spike, recovery, and linearity protocol for validating untested samples of R&D systems. The average recovery was 106.1% and 117.7% and the average linearity was 94.9% and 110.1% for PPROM and PTL, respectively. The range of the VDBP standard curves was 156.25–10000 pg/mL. Prior to measurement, CVF samples were initially diluted 500-fold with DuoSet assay diluents and then assayed for VDBP levels. Five CVF samples with VDBP levels that were undetected at a 500-fold dilution were diluted at 50-fold for final analysis. In the samples with VDBP levels that were lower than the lowest point on the standard curve, the lowest detected values were used for analysis. All the samples were assayed in duplicate. The intra- and inter-assay coefficients of variation were each <10%.

The management of women with PTL or PPROM has been previously described in detail [8, 16]. In short, prophylactic antibiotics were administered in all women with PPROM; however, the choice of antibiotics was left at the discretion of each attending obstetrician. Ampicillin and azithromycin (clarithromycin or erythromycin) were the main antibiotics used. Tocolytic therapy (i.e., magnesium sulfate, ritodrine, or atosiban) was administered at the discretion of the attending obstetrician. Antenatal corticosteroids were administered between 24 and 34 weeks of gestation. Intra-amniotic infection was defined as the presence of a positive AF culture result for microorganisms. Imminent preterm delivery was defined as SPTD within 48 hours after sampling. Histologic chorioamnionitis and funisitis were diagnosed according to previously published criteria [17]. Clinical chorioamnionitis was defined according to the criteria proposed by Gibbs et al. [18] Medications such as antibiotics, corticosteroids, and tocolytics were started after sampling.

Statistical analyses were performed with SPSS for Windows version 21.0 (IBM SPSS Inc., Chicago, IL, USA). The Shapiro-Wilk test was used to assess whether data in the groups were normally distributed. Continuous data were analyzed using the Student’s *t*-test or Mann-Whitney U test and were expressed as mean and standard deviation (SD). Categorical data were compared using the *χ*^2^-test or Fisher’s exact test and were presented as the percentage and number of patients in the groups. A multiple logistic regression analysis was then used to examine the independent relationship of the VDBP level in CVF to the outcome measures after controlling for baseline variables, with a *P* value < 0.1 in univariate analysis. Receiver operating characteristic (ROC) curves for the prediction of the outcome measures were generated for VDBP in CVF and used to identify the optimal cutoff value (maximizing the sum of sensitivity and specificity) for VDBP. The sampling-to-delivery interval was assessed using the Kaplan-Meier analysis and was compared between the groups using the log-rank test. Women who delivered preterm due to maternal or fetal indications and those who were lost to follow-up were included in this analysis, with a censoring time equal to the sampling-to-delivery interval. All reported *P* values were two-sided, and *P* values <0.05 were considered statistically significant.

## Results

During the study period, a total of 251 women with PTL (n = 148) or a diagnosis of PPROM (n = 103) met the eligibility criteria and were included in the final analysis. The mean (SD) gestational ages at sampling were 29.9 (2.9) weeks for the PTL group and 30.3 (3.1) weeks for the PPROM group (*P* = 0.140), while the mean (SD) gestational ages at delivery were 35.1 (4.3) weeks for the PTL group and 31.9 (2.6) weeks for the PPROM group (*P* < 0.001). Positive AF culture results were obtained in 9.4% (14/148) of the women with PTL and in 42.7% (44/103) of the women with PPROM. Table 1 shows the types of microorganisms isolated from the AF of women with PTL and PPROM. Polymicrobial invasion was present in 12 cases [85% (12/14)] for the women with PTL and in 26 cases [59% (26/44)] for the women with PPROM. For the analyses of the relationship between SPTD within 48 hours and the covariates, we excluded 12 patients because of iatrogenic deliveries (two for PTL and three for PPROM) and transfer to another hospital within 48 hours (three for PTL and four for PPROM). SPTD within 48 hours occurred in 18% (26/143) of women with PTL and 27% (28/96) of women with PPROM.

**Table 1:** Types of microorganisms isolated from the amniotic fluid of women with preterm labor and preterm premature rupture of membranes.

Microorganism	Number of cases
**Preterm labor and intact membranes**	
*Ureaplasma urealyticum*	14
*Mycoplasma hominis*	12
Gram-positive cocci	1
**Preterm premature rupture of membranes**	
*Ureaplasma urealyticum*	34
*Mycoplasma hominis*	27
*Streptococcus viridans*	3
Gram-positive cocci	2
*Candida* species	1
*Staphylococcus aureus*	1
*Haemophilus influenzae*	1
*Streptococcus agalactiae*	1
*Escherichia coli*	1
*Lactobacillus* species	1
Gram-negative rods	1

VDBP was detected in the CVF of 246 (98%) of the 251 women, and all five women with undetectable levels of VDBP in the CVF sample had PTL without intra-amniotic infection. Among the women with PTL, the CVD VDBP level was not correlated with gestational age at sampling both in the whole study group (r = -0.038, *P* = 0.642) and in the subgroup presenting without infection (r = -0.007, *P* = 0.938). In terms of the correlation between the CVF VDBP level and gestational age at sampling in the PPROM group, the results were the same.

Table 2 shows the demographic and clinical characteristics stratified according to the results of the AF culture and the occurrence of SPTD within 48 hours in the group of women with PTL. The women in the PTL group with intra-amniotic infection had a significantly lower median gestational age at sampling, higher median serum CRP level, and higher rate of administration of antibiotics and corticosteroids than those in the PTL group without intra-amniotic infection. The median CVF VDBP level was significantly higher in the women with intra-amniotic infection than in those without intra-amniotic infection. The results of univariate analysis with regard to SPTD ≤48 hours vs. >48 hours showed higher median serum CRP and CVF VDBP levels, a lower rate of nulliparity, and a higher rate of positive AF culture in the women who delivered preterm within 48 hours than in those who delivered preterm after 48 hours. Moreover, the rates of administration of corticosteroids and clinical chorioamnionitis also showed a tendency to be related to the occurrence of SPTD within 48 hours (*P =* 0.062 and *P =* 0.085, respectively), although they did not show any statistical significance.

**Table 2:** Clinical characteristics of the study population according to the results of the amniotic fluid culture and occurrence of delivery within 48 hours of sampling in women with preterm labor and intact membranes.

	Intra-amniotic infection		P values	Spontaneous preterm delivery after sampling		P values
Characteristics	Absent (n = 134)	Present (n = 14)		≤48 h (n = 26)	>48 h (n = 117)	
Maternal age (year)	32.0 ± 4.2	31.4 ± 3.9	0.398	32.0 ± 3.1	31.9 ± 4.5	0.885
Nulliparity	67% (90/134)	79% (11/14)	0.549	46% (12/26)	74% (86/117)	0.007
Gestational age at sampling (weeks)	30.1 ± 2.9	27.9 ± 2.4	0.003	30.0 ± 2.8	29.9 ± 2.9	0.915
Gestational age at delivery (weeks)	35.8 ± 3.8	29.9 ± 4.0	<0.001	30.1 ± 2.8	36.6 ± 3.5	<0.001
Serum CRP (mg/dL)	0.9 ± 1.8	3.3 ± 2.8	<0.001	2.8 ± 2.9	0.8 ± 1.4	<0.001
Cervicovaginal VDBP (μg/mL)	1.24 ± 1.46	2.43 ± 2.06	0.049	2.36 ± 1.85	1.14 ± 1.42	0.001
Positive AF culture				27% (7/26)	6% (7/117)	0.001
Use of tocolytics	93% (125/134)	100% (14/14)	1.000	96% (25/26)	93% (109/117)	1.000
Use of antibiotics	37% (50/134)	71% (10/14)	0.020	46% (12/26)	39% (46/117)	0.521
Use of antenatal corticosteroids	66% (88/134)	100% (14/14)	0.005	85% (22/26)	65% (76/117)	0.062
Clinical chorioamnionitis	1% (1/134)	14% (2/14)	0.024	8% (2/26)	1% (1/117)	0.085
Histologic chorioamnionitis^a^	37% (19/52)	85% (11/13)	0.004	69% (18/26)	32% (12/37)	0.004
Funisitis^a^	6% (3/52)	62% (8/13)	<0.001	27% (7/26)	11% (4/37)	0.176

CRP, C-reactive protein; VDBP, vitamin D-binding protein; AF, amniotic fluid.

Data are given as the mean ± standard deviation or % (n/N).

^a^Data for the histologic evaluation of the placenta were available in 65 (43.9%) of the 148 women because in 32 cases, the delivery took place at another institution and in 51 cases, histologic evaluation of the placenta was not performed because of our institutional policy that only the placentas in cases of preterm delivery are to be sent for histopathologic examination.

The results of univariate analysis in the group of women with PPROM are shown in Table 3. Similar to the results for intra-amniotic infection in the PTL group, low mean gestational age at sampling and high mean serum CRP and CVF VDBP levels were observed in the group of women with PPROM who had intra-amniotic infection. However, when SPTD within 48 hours was used as an outcome measure, the median CVF VDBP levels were not significantly different between women who delivered preterm within and after 48 hours.

**Table 3:** Clinical characteristics of the study population according to the results of the amniotic fluid culture and occurrence of delivery within 48 hours of sampling in women with preterm premature rupture of membranes.

	Intra-amniotic infection		P values	Spontaneous preterm delivery after sampling		P values
Characteristics	Absent (n = 59)	Present (n = 44)		≤48 h (n = 28)	>48 h (n = 68)	
Maternal age (years)	32.4 ± 4.0	33.3 ± 3.0	0.172	32.8 ± 2.8	32.6 ± 3.8	0.478
Nulliparity	54% (32/59)	46% (20/44)	0.378	46% (13/28)	53% (36/68)	0.562
Gestational age at sampling (weeks)	31.0 ± 2.9	29.3 ± 3.0	0.001	31.7 ± 2.0	29.6 ± 3.3	0.001
Gestational age at delivery (weeks)	33.0 ± 2.1(55)	30.3 ± 2.5(40)	<0.001	31.8 ± 1.9	31.8 ± 3.0(64)	0.460
Serum CRP (mg/dL)	0.6 ± 0.7	1.3 ± 1.4	0.009	1.1 ± 1.5	0.7 ± 0.9	0.120
Cervicovaginal VDBP(μg/mL)	2.20 ± 1.69	2.76 ± 1.66	0.042	2.15 ± 1.4	2.6 ± 1.8	0.417
Positive AF culture				57% (16/28)	37% (25/68)	0.067
Use of tocolytics	54% (32/59)	64% (28/44)	0.339	54% (15/28)	59% (40/68)	0.636
Use of antibiotics	92% (54/59)	100% (44/44)	0.070	90% (25/28)	99% (67/68)	0.073
Use of antenatal corticosteroids	83% (49/59)	91% (40/44)	0.384	79% (22/28)	91% (62/68)	0.090
Clinical chorioamnionitis	3% (2/59)	11% (5/44)	0.134	7% (2/28)	6% (4/68)	1.000
Histologic chorioamnionitis	43% (23/54)	75% (30/40)	0.002	57% (16/28)	56% (35/63)	0.888
Funisitis	22% (12/54)	38% (15/40)	0.106	29% (8/28)	29% (18/63)	1.000

CRP, C-reactive protein; VDBP, vitamin D-binding protein; AF, amniotic fluid.

Data are given as the mean ± standard deviation or % (n/N).

^a^Data for the histologic evaluation of the placenta were available in 94 (91.2%) of the 103 women because in eight cases, the delivery took place at another institution, and in one case, histologic evaluation of the placenta was not performed because of our institutional policy that only the placentas in cases of preterm delivery are to be sent for histopathologic examination.

The results of multivariate logistic regression analyses for each group of women with PTL and PPROM are shown in Table 4. In the group of women with PTL, logistic regression revealed that a high CVF VDBP level was independently and significantly associated with the intra-amniotic infection and imminent preterm delivery after adjustment for other confounders (for intra-amniotic infection: gestational age at sampling and maternal CRP; for SPTD within 48 hours: maternal CRP, parity, clinical chorioamnionitis, positive AF culture, and use of antenatal corticosteroids). However, in the group of women with PPROM, a high CVF VDBP level was not independently associated with intra-amniotic infection.

**Table 4:** Association between cervicovaginal vitamin D-binding protein and risks of intra-amniotic infection and delivery within 48 hours, as determined by logistic regression analyses in women with preterm labor and preterm premature rupture of membranes.

Variable	Intra-amniotic infection		Latency < 48 hours	
	P value	OR (95% CI)	P value	OR (95% CI)
**Preterm labor and intact membranes**				
Gestational age at sampling (weeks)	0.079	0.815 (0.649–1.024)		
Maternal CRP (mg/dL)	0.094	1.207 (0.968–1.503)	0.002	1.505 (1.155–1.961)
Cervicovaginal VDBP (μg/mL)	0.048	1.405 (1.003–1.968)	0.023	1.422 (1.049–1.927)
Nulliparity			0.002	0.170 (0.056–0.519)
Clinical chorioamnionitis			0.972	1.054 (0.059–18.901)
Positive amniotic fluid culture			0.306	2.285 (0.470–11.106)
Use of antenatal corticosteroids			0.220	2.379 (0.595–9.517)
**Preterm premature rupture of membranes**				
Gestational age at sampling (weeks)	0.039	0.860 (0.746–0.993)		
Maternal CRP (mg/dL)	0.014	1.838 (1.130–2.989)		
Cervicovaginal VDBP (μg/mL)	0.281	1.149 (0.893–1.479)		

OR, odds ratio; CI, confidence interval; CRP, C-reactive protein; VDBP, vitamin D-binding protein.

**Fig 1:**
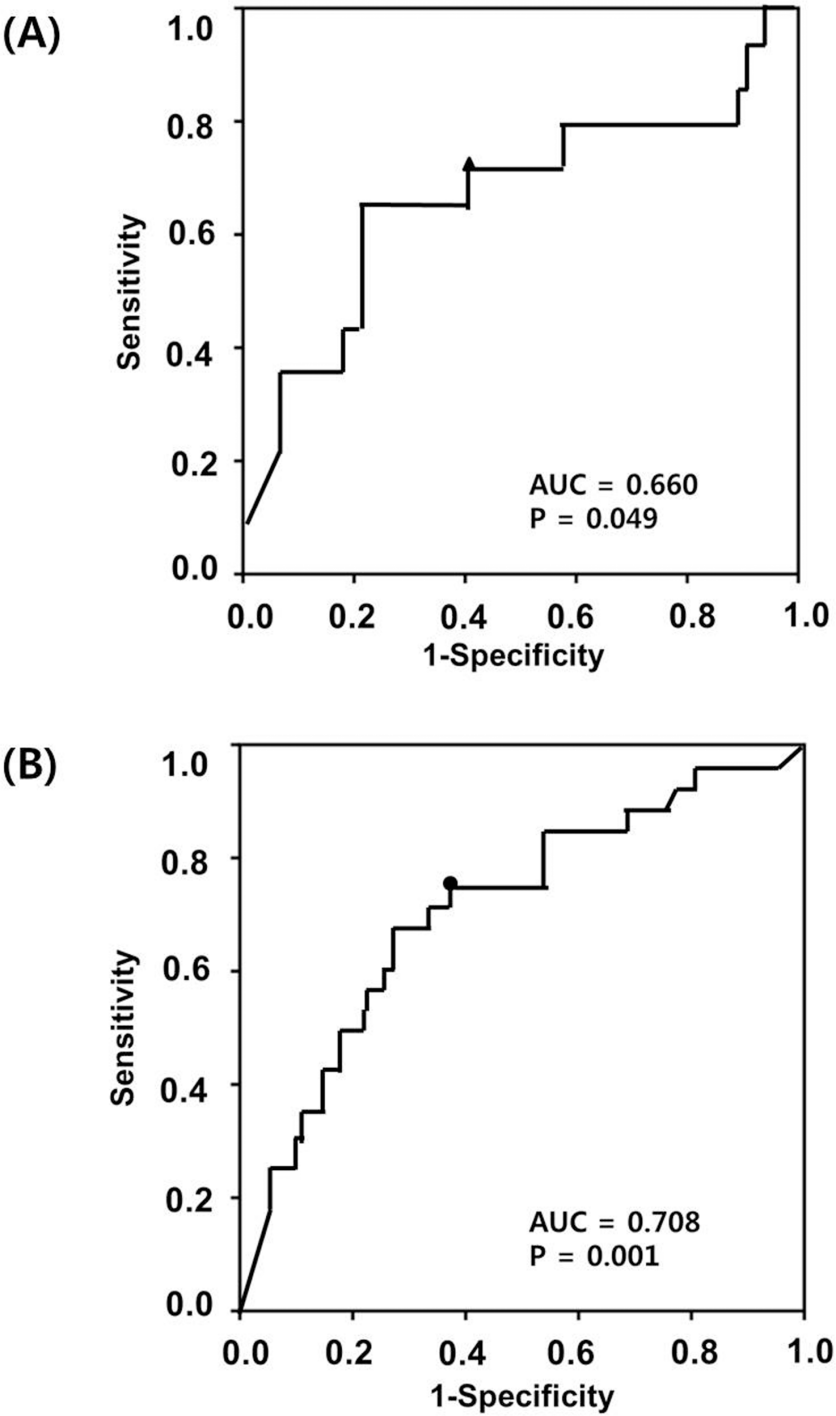
**Receiver-operating characteristic curves for vitamin D-binding protein in the cervicovaginal fluid** in the prediction of (A) intra-amniotic infection (area under the curve [AUC] 0.660, SE 0.092, *P* = 0.049), and (B) imminent preterm delivery within 48 hours (AUC 0.708, SE 0.056, *P =* 0.001) in women with preterm labor and intact membranes.

**Fig 2:**
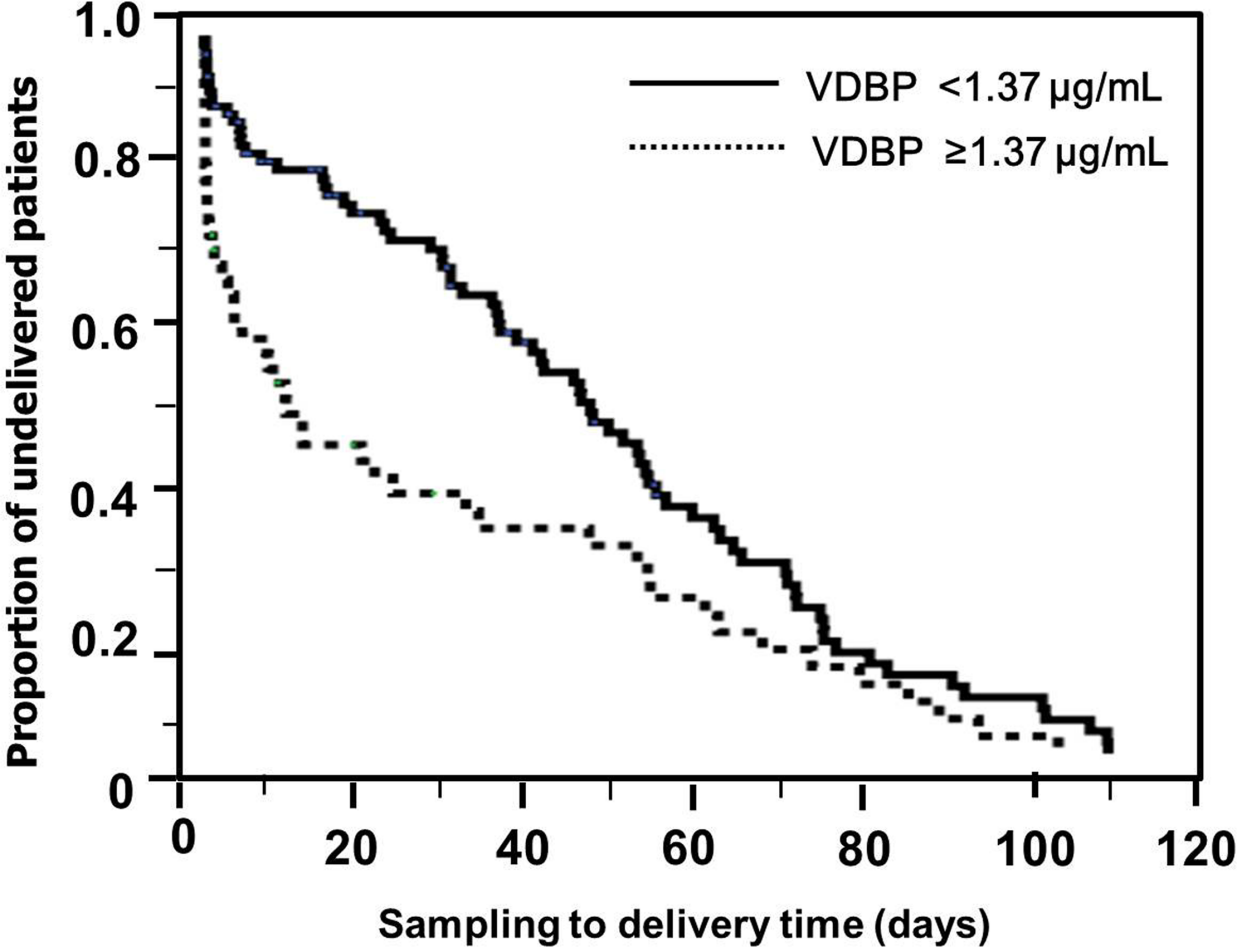
Kaplan-Meier survival estimates of the sampling-to-delivery interval according to the levels of vitamin D-binding protein (VDBP) in the cervicovaginal fluid of women with preterm labor and intact membranes (using 1.37 μg/mL as a cut-off) (median, 9.91 days [95% confidence interval (CI), 5.17–14.65] vs. 45.87 days [95% CI, 36.84–54.90]; *P* = 0.003).

Fig 1 displays the ROC curves for the CVF VDBP levels for predicting intra-amniotic infection and imminent preterm delivery in women with PTL. The areas under the curve (AUCs) of the CVF VDBP level for predicting intra-amniotic infection and imminent preterm delivery were 0.66 and 0.71, with cut-off values of 1.76 μg/mL [sensitivity of 64.3%, specificity of 78.4%, positive predictive value (PPV) of 23.6%, and negative predictive value (NPV) of 95.4%] and 1.37 μg/mL (sensitivity of 65.4%, specificity of 72.6%, PPV of 36.0%, and NPV of 89.4%), respectively. Fig 2 displays the Kaplan-Meier estimates of the sampling-to-delivery interval for the VDBP of ≥1.37 or <1.37 μg/mL. Comparison using the log-rank tests was significant for VDBP (*P* = 0.003).

The maternal demographic and clinical characteristics according to the membrane status (ruptured or intact membranes) are presented in Table 5. The women in the PTL group were younger and had a significantly higher rate of nulliparity and a lower prevalence of positive AF cultures compared to those in the PPROM group. The CVF VDBP levels were significantly higher in women with PPROM than in those with PTL, and this relationship remained significant after adjusting for maternal age, parity, and AF culture results (VDBP in CVF (μg/mL), odds ratio: 1.37; 95% confidence interval, 1.15–1.63, *P* < 0.001). In the subgroup of women without intra-amniotic infection, the CVF VDBP levels were significantly higher in women with PPROM than in those with PTL (mean ± SD: 2.20 ± 1.69 μg/mL vs. 1.232 ± 1.45 μg/mL, *P* < 0.001).

**Table 5:** Clinical characteristics of the study population according to the status of the fetal membrane (intact or ruptured membranes).

	Preterm labor and intact membranes (n = 148)	Preterm premature rupture of membranes (n = 103)	*P* value
Maternal age (years)	31.9 ± 4.2	32.8 ± 5.6	0.022
Nulliparity	68% (101/148)	51% (52/103)	0.005
Gestational age at sampling (weeks)	29.9 ± 2.9	30.3 ± 3.1	0.140
Gestational age at delivery (weeks)	35.1 ± 4.3	31.9 ± 2.6	<0.001
Serum CRP (mg/dL)	1.16 ± 2.02	0.86 ± 1.11	0.639
Cervicovaginal VDBP (μg/mL)	1.35 ± 1.56	2.44 ± 1.69	<0.001
Positive AF culture	9% (14/148)	43% (44/103)	<0.001
Use of tocolytics	94% (139/148)	58% (60/103)	<0.001
Use of antibiotics	41% (60/148)	95% (88/103)	<0.001
Use of antenatal corticosteroids	69% (102/148)	86% (98/103)	0.001
Clinical chorioamnionitis	2% (3/148)	7% (7/103)	0.097
Histologic chorioamnionitis	46% (30/65)	56% (53/94)	0.204
Funisitis	17% (11/65)	29% (27/94)	0.086

CRP, C-reactive protein; VDBP, vitamin D-binding protein; AF, amniotic fluid.

Data are given as the mean ± standard deviation or % (n/N).

## Discussion

The principal findings of this study are as follows: (i) in women with PTL, the CVF VDBP level independently predicts intra-amniotic infection and imminent preterm delivery; (ii) however, an elevated CVF VDBP level in women with PPROM was not independently associated with increased risks of either intra-amniotic infection or imminent preterm delivery; and (iii) CVF VDBP levels were significantly higher in women with PPROM than in those with PTL. This study confirms the findings of previous studies conducted in women with PTL by Liong et al. [13, 14] and Hitti et al. [11] and extends the findings to women with PPROM.

In accordance with the results of the current study, the previous longitudinal study by Liong et al. showed that VDBP is detectable in the CVF of all pregnant women during the second half of pregnancy, with no change in the CVF VDBP level between 20 and 35 weeks’ gestation [15], suggesting that VDBP is a physiologic constituent of the lower genital tract fluid. In addition, VDBP has been previously identified in the AF of pregnant women [19], and our preliminary experiment to determine the dilution ratio showed that VDBP levels in the AF without intra-amniotic infection were approximately 10 times higher than the levels in the CVF (supplementary materials). Similarly, the VDBP levels reported in pregnant maternal serum were 1000 times higher than those measured in the CVF in the current study [20, 21]. Thus, the most likely source for VDBP present in the CVF in the absence of infection or inflammation may be transudate from the maternal serum or AF.

The present study demonstrates that in women with PTL, CVF VDBP level was independently predictive of SPTD within 48 hours. These observations are consistent with the results of a previous study by Liong et al., who demonstrated that the CVF expression of albumin and VDBP increased significantly in women with symptoms of PTL who delivered preterm, compared with that of those who delivered at term [14]. In asymptomatic women, elevated CVF VDBP levels have been previously reported to be associated with spontaneous term labor, PTL, and the occurrence of PPROM [13, 15]. Similarly, a recent study by our group demonstrated that an elevated CVF VDBP level is independently predictive of SPTD at <32 weeks in asymptomatic women with cervical insufficiency or a short cervix [22]. Taken together, these observations suggest that VDBP may be implicated in the mechanisms of premature cervical remodeling and preterm birth. On the other hand, the AUC value for the ability of VDBP to predict SPTD within 48 hours was relatively small (0.71) in the present study, which indicates that the clinical usefulness of CVF VDBP is limited when used alone, and highlights the need to combine various predictors for preterm birth in light of its multifactorial and complex etiology. Therefore, further studies are warranted to explore the utility of VDBP levels in CVF in combination with other important clinical factors (e.g., fetal fibronectin level and sonographic cervical length) in the prediction of SPTD.

In contrast to the women with PTL, elevated VDBP levels analyzed in the CVF of women with PPROM were not significantly associated with an increased risk of SPTD within 48 hours. This discrepant result between PTL and PPROM suggests that the pathophysiology and molecular mechanisms associated with SPTD that occurs as a result of PPROM and PTL may differ, and reflects a different role of VDBP in the regulation of preterm parturition in the context of these two different disease entities.

In univariate analysis, we found that an elevated CVF VDBP level was associated with positive AF cultures in both PTL and PPROM. These observations confirm the findings of a previous proteomic study conducted in women with PTL by Hitti et al., which demonstrated that VDBP in vaginal fluid was expressed differently between women with and without intra-amniotic infection (defined as positive AF cultures and/or IL-6 >2 ng/mL) [11]. We also expanded the observations reported by Hitti et al. by demonstrating that this relationship is independent of other risk factors for intra-amniotic infection/inflammation in PTL, such as gestational age at sampling and serum CRP [23], according to multivariate logistic analysis. Collectively, these findings support the view that CVF VDBP may be implicated as a potential participant in the regulation of the host response to intra-amniotic infection. If so, what then is the plausible mechanism for the association between an elevated CVF VDBP level and positive AF cultures in PTL? The most likely mechanism of this association may be related to the potential acceleration of the transudative process of solutes crossing the inflamed or infected chorioamniotic membrane from the amniotic cavity into the CVF [24], which occurs in intra-amniotic infection. Indeed, the previous reports by Ma et al. [25] and Guha et al. [26] support this speculation. Ma et al. found VDBP to be expressed in the trophoblasts of normal placentas throughout pregnancy [25]. Guha et al. have shown that IL-6 can increase the synthesis of VDBP *in vitro* [26]. Alternatively, the CVF VDBP level can be significantly elevated in the context of the high local production of this protein in the cells of the cervix or vagina that occurs in response to vaginal or cervical infection associated with ascending infection (e.g., bacterial vaginosis) [27].

The results of the current study show that the CVF VDBP levels were significantly higher in women with PPROM than in those with PTL, even in the subgroup of cases without microbial invasion. These findings suggest that a mechanism associated with altered levels of VDBP in the CVF may contribute to fetal membrane remodeling, weakening, and rupture. This speculation was supported by reports indicating that VDBP is a potent activation factor for macrophage, neutrophils, and monocytes [28, 29] that secrete tumor necrosis factor, IL-1, IL-6, and chemokines and activate MMP-9, leading to potential fetal membrane weakening and rupture [30]. Another interpretation for this finding is that PPROM allows at least small quantities of AF to spill into the CVF; thus, the CVF VDBP levels along with the high VDBP levels in the AF may be elevated further in cases of PPROM.

Our study had several limitations. First, we lacked data on other potentially important tests that could contribute to the assessment of the risk of SPTD (i.e., fetal fibronectin and sonographic cervical length) [14, 31, 32]. Second, we did not use a molecular technique (i.e., PCR) to detect microbes or their DNA and/or RNA in AF, and thus cannot exclude the possibility of the presence of uncultivated microorganisms in the women with intra-amniotic infection. Third, the current study did not include information on confounding factors for the relationship between the VDBP level and outcomes, such as recent sexual intercourse, bacterial vaginosis, vaginal microbiology, and CVF albumin level, although these variables (except for CVF albumin level) have not been previously implicated as confounders of CVF VDBP levels [14, 15]. Fourth, the study was of a retrospective nature, which may lead to not correcting for major potential confounders despite conducting multivariate analysis. Fifth, this study was performed at a single center, which may limit the generalizability of our findings. Therefore, the results of this study need to be prospectively validated in a different, larger population. Sixth, the current study was limited by the fact that the samples were not randomly analyzed; thus, the significance of the findings, when used for population inference, may have an error to an unknown degree. Seventh, the fact that VDBP was assayed in stored samples after the development of the measured outcomes prevented any effect of the VDBP levels on the decision-making process regarding each case. Eighth, our study was limited by the fact that multiple testing corrections were not applied owing to the explorative nature of the study, which may lead to false-positive results. Finally, we did not obtain test results for lower genital tract microbiology, even though it may affect the CVF VDBP level according to a reported positive correlation between bacterial vaginosis and IL-1α and IL-1β levels [33]. Therefore, the current study did not clarify whether the origin of the elevated CVF VDBP levels is lower genital tract infection or intra-amniotic infection. The main strength of the study is that it is, to the best of our knowledge, the first study to characterize the change in the CVF VDBP levels in association with intra-amniotic infection and SPTD in a relatively large cohort of symptomatic women, where stratification was based on the fetal membrane status.

## Conclusions

In conclusion, we demonstrated that the level of VDBP in the CVF independently predicted intra-amniotic infection and imminent preterm delivery in women with PTL and that elevated CVF VDBP levels were not associated with increased risks of these two outcome variables in women with PPROM. Further studies are needed to determine the source of VDBP in the CVF and to elucidate the role of VDBP in maternal blood and AF as it relates to SPTD and intra-amniotic infection.

## Supporting information

S1 File Raw data for women with PTL. (SAV)

S2 File Raw data for women with PPROM. (SAV)

S3 File Raw data for comparison between the PTL and PPROM groups. (SAV)

S4 File Raw data for AF dilution test results for VDBP levels of the women with PTL and PPROM. VDBP levels in AF were undetected at a 10- fold, 100- fold, and 500-fold dilution, but were detected at 5000-fold dilution.
(XLSX)
